# Exploratory Temporal and Spatial Analysis of Myocardial Infarction Hospitalizations in Calgary, Canada

**DOI:** 10.3390/ijerph14121555

**Published:** 2017-12-11

**Authors:** Xiaoxiao Liu, Stefania Bertazzon

**Affiliations:** Department of Geography, University of Calgary, Calgary, AB T2N1N4, Canada; bertazzs@ucalgary.ca

**Keywords:** myocardial infarction, temporal analysis, seasonal trend decomposition, spatial analysis, hot spot analysis, urban air pollution, Canada

## Abstract

Spatial and temporal analyses are critical to understand the pattern of myocardial infarction (MI) hospitalizations over space and time, and to identify their underlying determinants. In this paper, we analyze MI hospitalizations in Calgary from 2004 to 2013, stratified by age and gender. First, a seasonal trend decomposition analyzes the seasonality; then a linear regression models the trend component. Moran’s I and hot spot analyses explore the spatial pattern. Though exploratory, results show that most age and gender groups feature a statistically significant decline over the 10 years, consistent with previous studies in Canada. Decline rates vary across ages and genders, with the slowest decline observed for younger males. Each gender exhibits a seasonal pattern with peaks in both winter and summer. Spatially, MI hot spots are identified in older communities, and in socioeconomically and environmentally disadvantaged communities. In the older communities, higher MI rates appear to be more highly associated with demographics. Conversely, worse air quality appears to be locally associated with higher MI incidence in younger age groups. The study helps identify areas of concern, where MI hot spots are identified for younger age groups, suggesting the need for localized public health policies to target local risk factors.

## 1. Introduction

It is well known that cardiovascular disease is a leading public health concern, contributing to 30% of global mortality [1,2]. It is one of the top two leading causes of death in Canada [3]. Among 17 million deaths caused by cardiovascular disease by 2015, as many as 7.6 million were attributed to coronary heart disease [4]. Myocardial infarction (MI), commonly known as heart attack, is one of the five main manifestations of coronary heart disease [1]. Spatial analysis in myocardial infarction provides a strategy to address spatially varying incidence patterns and to investigate the contextual factors surrounding it. For example, a study conducted in Quebec, Canada, reported the existence of spatial variability of hospitalization rates due to ischemic heart disease for both men and women [5]. With kernel density analysis and clustering methods, Majlund et al. 2016 examined the geographical patterns of acute myocardial infarction in Denmark and found that people who lived in the identified MI clusters featured a markedly lower socioeconomic status than those living outside the clusters [6]. A more recent study conducted at the health region scale (health regions are administrative areas of interest defined by the provinces. Most of them are usually organized along geographic boundaries) in Canada shows a statistically significant spatial autocorrelation (i.e., spatial clustering) (See Section 2.3) of MI hospitalizations for both genders, which suggests that the highest MI hospitalization rates tend to be clustered in the eastern regions of Canada, especially in Ontario, Quebec, and New Brunswick [7]. To date, most of the Canadian studies analyzing MI spatial patterns are conducted at the national, provincial, or health region level, with little research reported at city level [7].

Temporal analysis helps understand the evolving nature of health outcomes over time, identify long term trend and change in the frequency of disease occurrence. Researches typically consider two types of analysis: trend analysis and seasonality. Trend analysis has been conducted in many countries and results are consistent among studies [8,9,10], showing that mortality due to cardiovascular disease has declined in recent years. In Canada, the hospitalization rate for myocardial infarction decreased by 9.2% from 1994 to 2004 [11]. In the province of Ontario, the overall hospitalization rate for acute myocardial infarction declined by 36.7% for males and 39.0% for females from 1994 to 2014. People aged 20 to 49 experienced the lowest decline in MI rate over 20 years, whereas people aged between 65–74 years old show the largest decline rate for both males and females [12].

With respect to seasonality, MI generally tends to exhibit a distinct seasonal pattern with a peak in winter which is consistent among studies [13,14]. This relationship was observed for a variety of climates. For example, a study performed in the greater Athens area confirmed the negative association between MI hospital admission and mean daily temperature, suggesting a 5% increase in hospitalizations with one degree decrease in temperature [15]. However, the degree of decline and seasonality are not consistent through age and gender, because the prevalence of cardiovascular risk factors may vary across age groups and genders [8,16]. In addition, different age and gender groups may receive different benefits from improved MI management [8]. The inconsistency among groups suggests that there is a need for more research on the spatial and temporal trends of MI for each age and gender population. Spatial and temporal analyses are critical to investigate the mechanisms underlying past trends in MI hospitalizations and to provide evidence to evaluate progress in mitigating MI. They provide information for health system planners, policy makers as well as clinicians to make decisions about strategic planning, prioritization of health policy, and allocation of limited health system resources [17].

This study presents an exploratory analysis for detecting the temporal and spatial variations of MI hospitalizations in Calgary, Canada. The purpose of the study is a descriptive, rather than a confirmatory analysis. A seasonal trend decomposition is applied to decompose the original standardized MI hospitalization series into three components: seasonal, trend and remainders. The trend component is modeled with a linear regression against its corresponding time points; the seasonal component provides a way to detect the presence of seasonality; and the linear regression identifies the long-term linear trend. Spatial autocorrelation explores various spatial scales to determine the one where significant spatially clustered patterns can be detected. Finally, hot spot analysis is conducted for detecting spatial hot spots of MI within the city of Calgary.

## 2. Materials and Methods

### 2.1. Study Area

**Figure 1:**
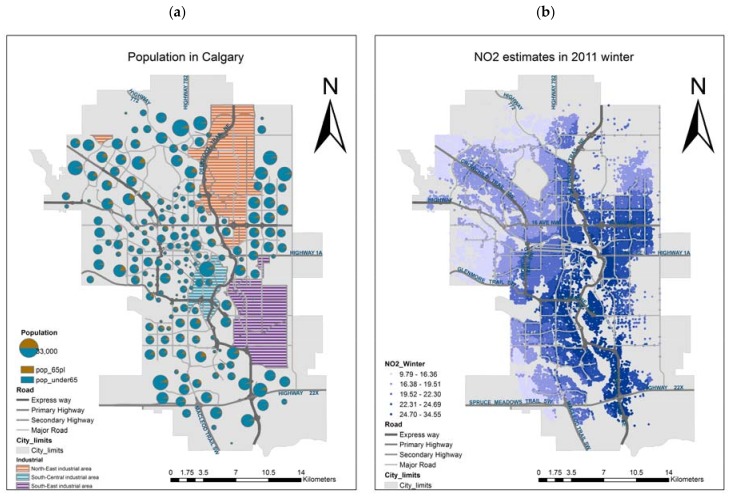
Population distribution (**a**), and air pollution (**b**) in Calgary.

With a population of approximately 1.2 million, the metropolitan area of Calgary covers a land area of 825.56 km^2^. It is a relatively young city, with a median age of 36.9 years in 2016 [18]. The communities with larger population are mainly located in the suburban areas; conversely, people over 65 tend to live in communities close to the downtown core, in the northwest, and in the southwest (Figure 1a). All subjects gave their informed consent for inclusion before they participated in the study. The study was conducted in accordance with the Declaration of Helsinki, and the protocol was approved by the Ethics Committee of University of Calgary (REB14-0158_REN3). This spatial pattern suggests that a high proportion of younger people, still in working age, live in suburban areas, where long commutes are associated with heavy traffic.

**Figure A1:**
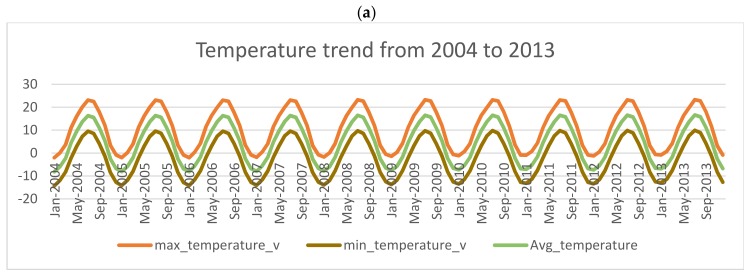

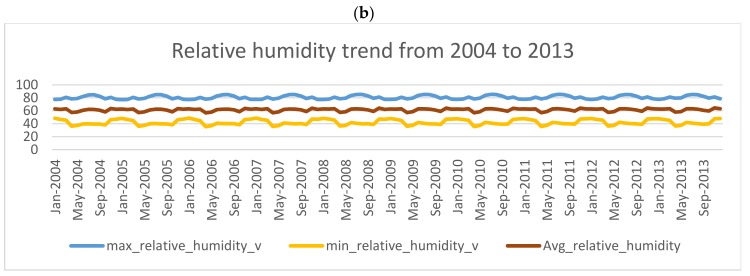
Time series of monthly temperature (**a**), relative humidity (**b**).

Located in the Canadian province of Alberta, approximately 80 km east of the Rocky Mountains, at an average elevation of 1000 m, Calgary’s prevailing winds blow from the west and from the north. The west wind blowing into Alberta over the Rocky Mountains brings warm and moist air from the Pacific Ocean, whereas the strong north winds carry cold and dry Arctic air [19]. Due to the prevailing west and north wind and high latitude (51.04 N), Calgarians enjoy a mostly sunny weather with strong seasonal differences. The hottest month is July, with an average temperature ranging from 9.8 to 23.2 degrees Celsius, whereas the coldest month is February, featuring average temperatures ranging from −0.9 to −13.2 degrees Celsius [20]. The climate trend in Calgary was quite stationary from 2004 to 2013. Monthly temperature peaked in July, with the lowest values in January. With respect to relative humidity, highest minimum humidity occurred in the warm months from June to August, and lowest in the cold months from December to January. Please see Figure A1 for detailed time series.

The three major industrial areas are the north-east industrial area, the south-central industrial area, and the south-east industrial area (Figure 1a). As a vast metropolitan city with a large traffic volume, transportation is the main contributor to nitrogen oxides (NO_x_), carbon oxide (CO), and fine particulate matter (PM_2.5_) emissions in Calgary [21]. Air pollution studies in Calgary identify the most highly polluted areas of NO_2_ concentrations (ppb) (Figure 1b) in the east, downtown core and along the major traffic corridors [22,23].

### 2.2. Data

Population and demographic data for the city of Calgary are available through the Calgary Civic Census [24]. The Calgary Civic Census collects dwelling numbers and population counts on an annual basis since 1958, usually beginning on 1 April each year. In addition, gender and age distribution data are collected citywide in the years when the Federal Census is taken, that is, the years ending in 1 and 6, plus an extra collection in between census years. As a result, age and gender data are available, for the period of interest, for the following years: 2004, 2006, 2009, 2011 and 2014. Age is grouped into the following categories: 0 to 4, 5 to 14, 15 to 20, 20 to 24, 25 to 34, 35 to 44, 45 to 54, 55 to 64, 65 to 74 and 75+.

Myocardial infraction hospitalization data were provided by the Alberta Provincial Project for Outcome Assessment in Coronary Heart disease (APPROACH) [25], which collects and processes information to improve cardiac care in Alberta, Canada. Among 12,389 patients with acute coronary syndrome from 2004 to 2013, 6142 persons aged between 20 and 99 years were admitted to a hospital with myocardial infarction as either primary or secondary diagnosis. MI data were considered in different ways by the temporal and spatial analysis, respectively. For the temporal analysis, individuals from this set were grouped by age and gender, consistent with the demographic data, and result in a total of 10 groups: Females (20 to 44, 45 to 54, 55 to 64, 65 to 74 and 75+), and Males (20 to 44, 45 to 54, 55 to 64, 65 to 74 and 75+) [8,11,24]. Monthly age and gender specific rates were calculated for each month. Monthly MI hospitalizations for each age and gender group were first counted, then divided by the population for each age and gender in the corresponding month, as reported by 2014 Calgary civic census, and finally multiplied by the age and gender stratified population based on the 2011 federal census data. For the spatial analysis, MI data were reorganized at dissemination area level, which is the smallest spatial unit where social economic and demographic data is collected in Canada [26]. MI hospitalizations, at each DA, were divided by population and multiplied by 100.

### 2.3. Methods

#### 2.3.1. Temporal Analysis

Seasonal trend decomposition was applied for decomposing the time series of MI into three temporal components: trend, seasonal and reminder [27,28]. The trend component accounts for the low frequency variation in the data, and the nonstationary long-term changes over time. It is not necessarily linear [29]. The seasonal component represents the variation in the data caused by seasonal frequency. The remaining component, i.e., remainder, is the residual variation, not captured by either the trend or seasonal component. Loess, a locally weighted regression by local fitting, was used for a sequence of smoothing operations in this procedure [27]. The procedure first defines a local temporal neighborhood; then, each time point in the neighborhood is assigned a weight based on its distance to the time point to be estimated [30]. The trend component isolated through the decomposition is fitted by means of a linear regression model, which uses time itself as the single independent variable [31]. Linear regression captures the stationary long term trend, without taking the local variations around the trend into account. The ordinary least squares (OLS) method is used for the estimation of the unknown trend coefficient by minimizing the sum of the squared differences between the observed data and the values predicted by the linear function.

#### 2.3.2. Spatial Analysis

Moran’s I is employed for defining spatial scale with significant spatial autocorrelation, and hot spot analysis for identifying statistically significant hot spots. Moran’s I is a basic measurement of spatial autocorrelation [32,33]. The null hypothesis is that the distribution of values in the study area follows a random pattern. The Moran’s I index is defined as:

(1) $$I=\frac{n\sum_{i=1}^{n}\sum_{j=1}^{n}v_{ij}(x_{i}-\mu )(x_{j}-\mu )}{\sum_{i=1}^{n}\sum_{j=1}^{n}v_{ij}\sum_{i=1}^{n}{\left(x_{i}-\mu \right)}^{2}}$$

where n is the number of spatial units indexed as *i* and j. μ is the mean of samples. $v_{ij}$ is the spatial weight function among locations iandj. It can be defined as the inverse of distance among locations. The closer the points are, the higher the weight they are assigned. Alternatively, the weight function can be defined with a fixed distance threshold. Points without the neighborhood are given the weight of zero. Researchers have reported many other ways defining spatial relationship. Generally, basic understanding of the phenomenon in question is required for selecting the proper spatial weights function [34].

Moran’s I value ranges from −1 to 1. A high positive Moran’s I index indicates a clustered spatial pattern with high values surrounded by high values, or low values surrounded by low values. Conversely, a high negative Moran’s I index suggests a negative spatial autocorrelation. The spatial pattern is characterized by high values surrounded by low values, or low values surrounded by high values. Moran’s I values close to 0 indicate absence of spatial autocorrelation. The spatial pattern is random; that is, there is no systematic association of high and low values. Moran’s I can be transformed into a Z score to test the statistical significance of spatial autocorrelation.

Incremental spatial autocorrelation calculates Moran’s I with difference distances among locations and the index is graphed against the corresponding Z scores. The peak Z score suggests that a statistically significant spatial autocorrelation is observed at the corresponding distance in the study area. It is helpful for understanding the appropriate spatial scale of analysis in question [35].

Hot spot analysis detects clusters of both high values (hot spots) and low values (cold spots). A hot spot occurs where a high value of a variable is surrounded by other high values. It is calculated with the Getis-Ord Gi* statistic [36,37] for each location in the study area. The underlying null hypothesis is that the association of values within the defined neighborhood follows a random distribution drawn from the whole sample. It is defined as follows:

(2) $$G_{i}(d)=\frac{\sum_{j=1}^{n}w_{ij}(d)x_{j}}{\sum_{j=1}^{n}x_{j}}$$

where d is the distance between locations i and j; n denotes the number of spatial unites in the study area; $w_{ij}$ denotes the spatial weight function. Given the location of interest i and a radius distance from point j, the values located in the neighborhood are added based on the predefined spatial weight matrix which assigns higher weight to closer points. Alternatively, with a predefined distance band, points within the neighborhood are assigned a weight of one and those outside of the threshold are assigned zero [36]. By comparing the local sum of values in the neighborhood and the expected global sum, the Gi* statistic is calculated and standardized as a Z score indicating the statistical significance of the cluster. Higher local sums of values in the neighborhood suggest a higher Gi* statistic, indicating that the variable tends to be clustered with higher values (hot spot), whereas lower Gi* indicates a cluster of lower values (cold spot). Positive Z scores larger than the critical value indicate statistically significant hot spots, while, symmetrically, large negative Z scores indicate statistically significant cold spots [36].

## 3. Results

**Figure 2:**
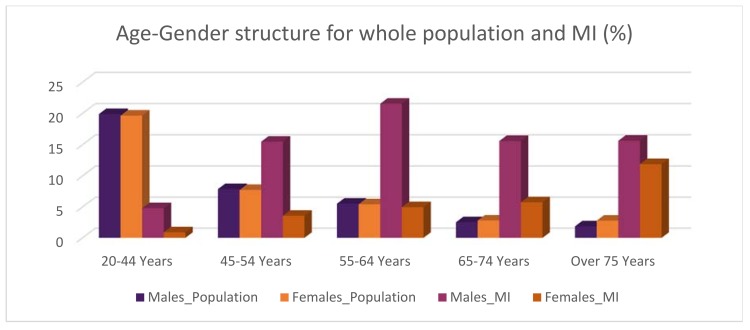
Age-gender structure for total population and myocardial infarction (MI) population in Calgary.

The total population of Calgary in 2014 was 1,195,194. Compared with 933,495 in 2004, this difference represents an increase of 28% over ten years [24], with an average annual increase of 2.8%. In 2014, the male population was 602,685, almost equal to the female population of 592,509. People aged 25 to 34 were the largest population group, totalling 210,851, followed by people aged 35 to 44, and then by the 45 to 54 age group. Therefore, the population of Calgary exhibits fairly standard traits, with females and males making almost equal contributions to the total population, and the population of each age group decreasing with advancing age (Figure 2). Conversely, the contribution of gender and age groups to the MI population exhibits a very different pattern, shown in Figure 2. Males account for 73% of the 6141 MI cases, that is, 2.7 times higher than females (27%), indicating that males tend to experience a higher MI risk than females. With respect to age groups, “males 55 to 64” leads the way with 21.54% of total MI among male groups, followed by “males over 75” (15.62%), by “males 65 to 74” (15.57%), and then by “males 45 to 54” (15.47%). Among female groups, “females over 75” features the highest contribution, with a percentage of 11.87, followed, in their order, by each younger age group. In other words, older females are likely to be in higher risk of MI than younger groups.

A whole 15.62% of the MI population is constituted by “males over 75”, while this age group constitutes only 1.86% of the background population, meaning that the contribution of “males over 75” to the MI population is 8.4 times larger than its contribution to the background population. People aged over 65 of both genders contribute around 50% of MI population, suggesting MI population features a larger proportion of older people than the background population (11%).

### 3.1. Seasonal Trend Decomposition

**Figure 3:**
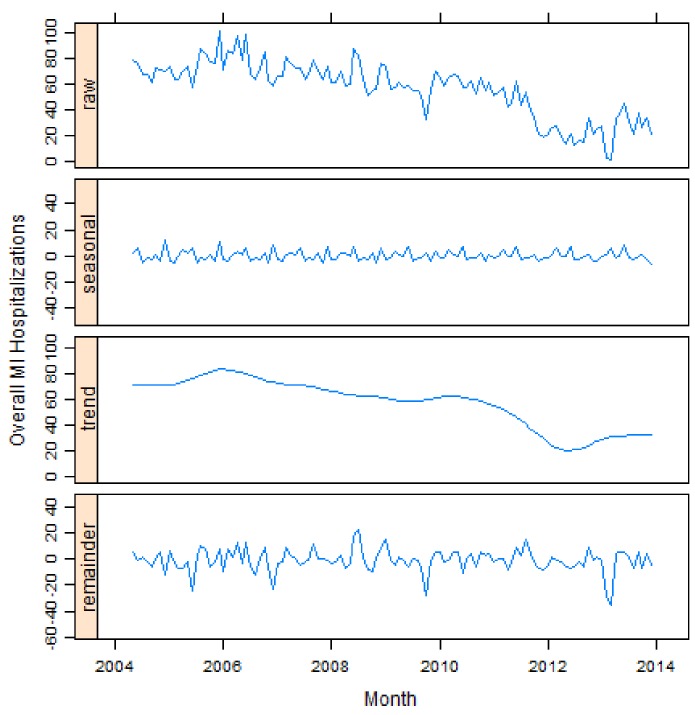
Seasonal trend decomposition plots for overall MI groups.

During the period between May 2004 and December 2013, all ten groups show a clear declining trend over time. Generally, the highest MI incidence occurred during the coldest months of 2005, while the lowest occurred in 2012 (Figure 3) (according to APPROACH personnel, it is likely that the drop in MI observed in 2012 is partially associated with recording issues, i.e., lacking or inconsistent reporting during that period).

**Figure A2:**
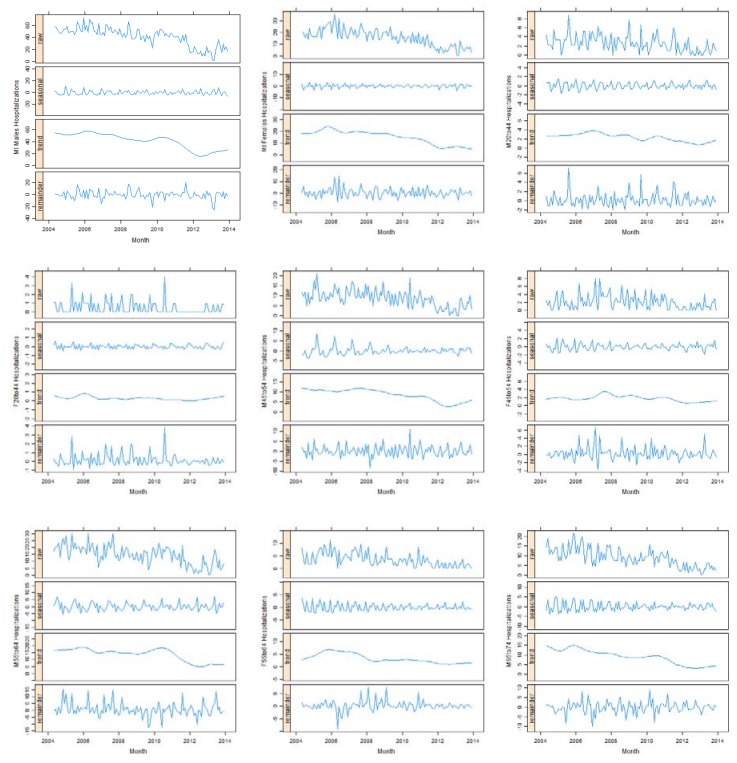

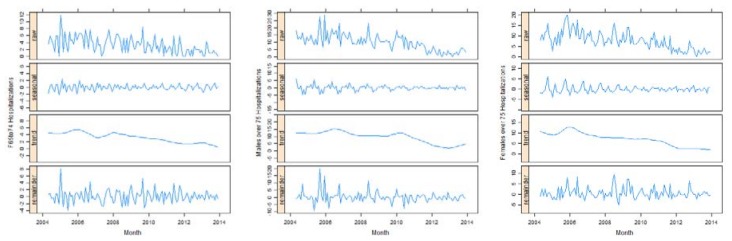
Seasonal trend decomposition for all age-gender specific groups.

The results of the seasonal trend decomposition for overall MI are shown in Figure 3. All other age and gender stratified MI groups are listed in Figure A2. The first panel of Figure 3 is the raw MI incidence, the second panel is the seasonal component, the third panel is the trend component, and the fourth panel is the remainder (i.e., residual) component. The seasonal component panels present a roughly yearly periodic pattern. The trend component panels display a slightly declining trend over the ten years.

**Figure 4:**
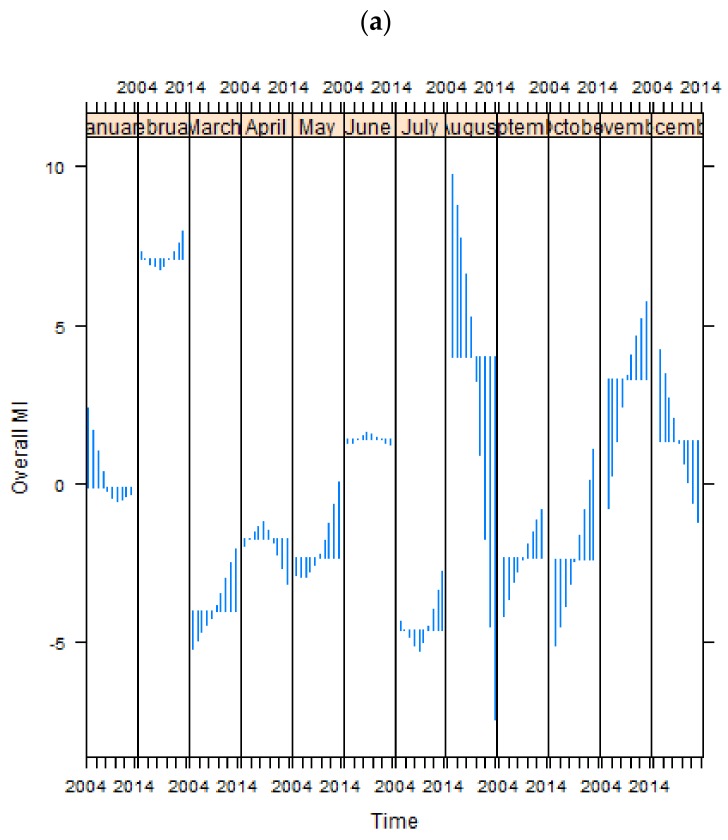

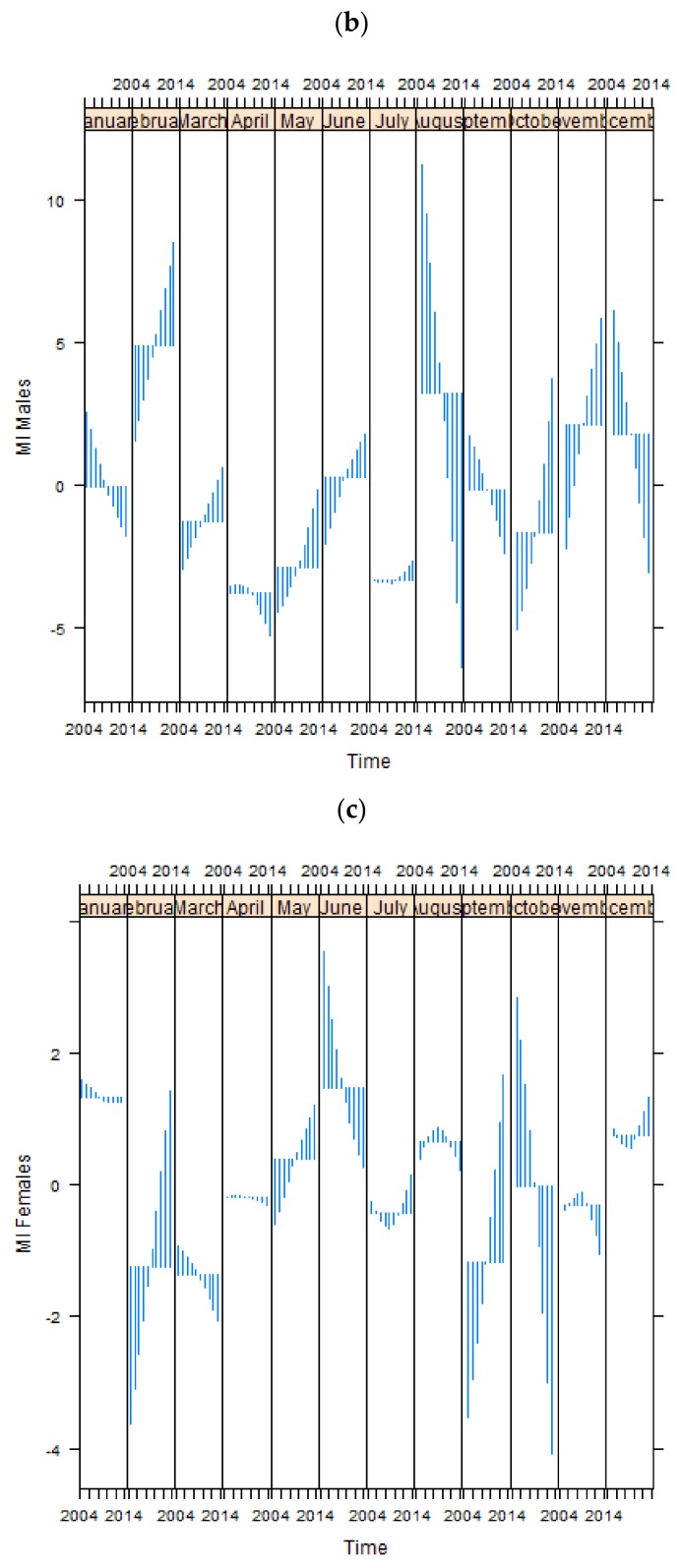
Cycle-subseries plots for (**a**) overall MI, (**b**) males and (**c**) females.

In order to analyze seasonal patterns in greater detail, each seasonal component was graphed against time, with the horizontal line indicating the average of MI hospitalizations in the same month over ten years and the vertical lines indicating the difference between mean and recorded MI hospitalization in each month (Figure 4). Overall, incidence exhibits some regularities: higher incidence occurs in the first months of the year, i.e., the coldest winter months; rates decline through the late winter and spring, to peak again in the summer months; a similar pattern is observed in the late-summer to early-winter months. The winter peak is generally higher than the summer one. The overall MI plot shows that the highest average of MI cases was recorded in February, followed by August (Figure 4a). The MI incidence for overall males (Figure 4b) peaked in February as did the overall MI population, likewise followed by August. Conversely, for overall females (Figure 4c) the peak time was in June, followed by a second one in December through January. Generally, warmer months show a larger interannual seasonal variation than the other months.

**Figure A3:**
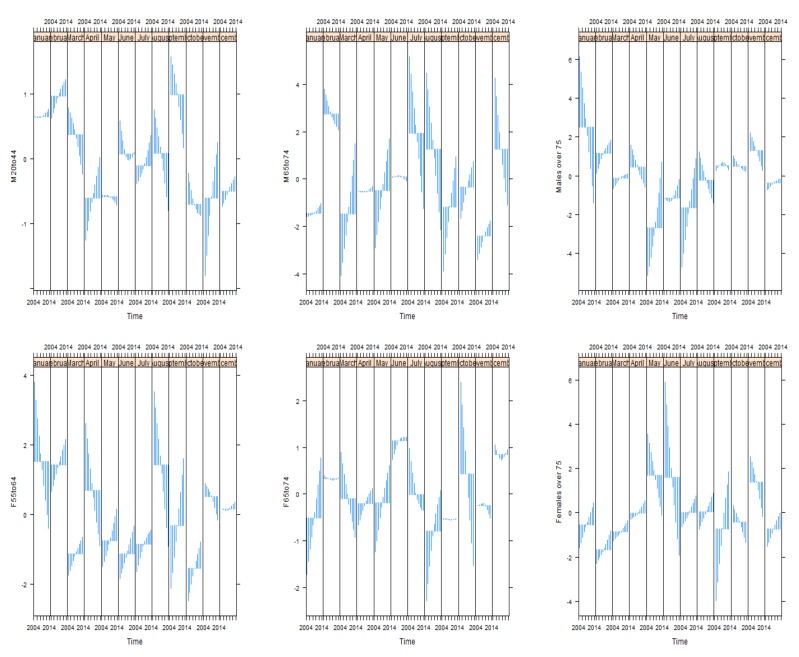
Cycle-subseries plots for all age-gender specific groups.

Most female age groups show a similar seasonality (see Figure A3). For “females 20 to 44”, “females 45 to 54” and “females 55 to 64”, the highest peak is in both summer (August) and winter (January and February), though the summer peak is slightly higher than the winter peak. For older women, i.e., “females 65 to 74” and “females over 75” summer and winter peaks occur somewhat earlier in each season, with the highest MI incidence happening in June, followed by the secondary peak around November and December.

Though males and females tend to experience high risk of MI in both winter and summer, males are more likely to develop MI in winter than females, while females are at greater risk in the summer. However it is not clear if the difference between summer and winter peaks is statistically significant. Generally, both winter and summer months show a larger variation around the mean than other months.

### 3.2. Linear Trend of MI Hospitalizations

According to the decomposed trend component plots (see Figure A2), a gradually declining trend with some local variations is observed for most groups, with the exception of the younger groups. For the larger MI groups (i.e., four male groups and two female groups), the MI trend components were fitted by a linear regression respectively, with time as the single explanatory variable. According to this analysis, most groups exhibit a statistically significant declining trend (Table 1).

All of the trend coefficients are highly statistically significant, as shown by t values ranging from −14.14 to −32.33 (*p*-value = 0.00), indicating that the null hypothesis of a zero trend coefficient can confidently be rejected.

The downward monthly change rates range from −0.04 to −0.11, suggesting the degree of decline varies across age and gender groups. Males show a monthly decreasing rate of −0.35, which is 2.33 times higher than females (−0.15). “Males 55 to 64” features the largest declining rate (−0.11), followed by “males 65 to 74” (−0.10), “males over 75” (−0.10), and “males 45 to 54” (−0.08). Generally, the MI incidence in younger males declines at a slower pace than older males. The coefficients of determination [38] range from 0.64 to 0.90, suggesting 64% to 90% of the variance in the MI variables are explained by the fitted linear model. Among the female groups, “females over 75” shows the sharpest decreasing rates followed by “females 65 to 74”. The coefficients of determination are 0.86 and 0.88, respectively, suggesting a good model fit.

### 3.3. Spatial Analysis

**Figure 5:**
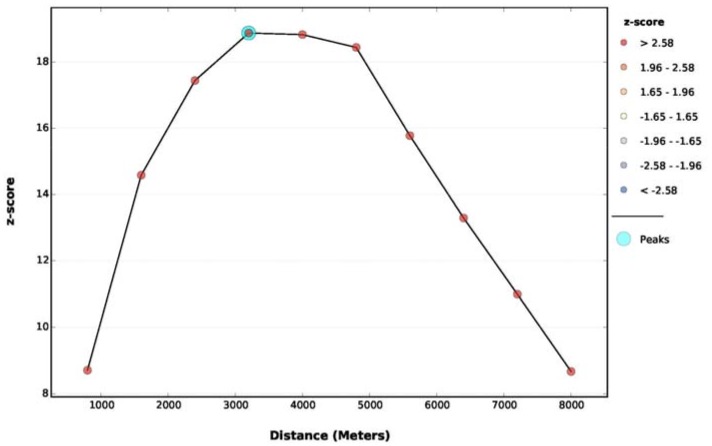
Spatial autocorrelation by distance for overall MI.

Moran’s I is run over different spatial distances, measuring the intensity of spatial clustering at each distance. The incremental spatial autocorrelation plot shows that the Z score increases along The plot, therefore, suggests that the highest cluster intensity is observed in the 3200–4000 m interval. However, the maximum distance observed for the nearest neighbors is 3620 m, indicating that, below this distance, there exist observations without neighbors, which may invalidate the significance of the corresponding results. Therefore, a distance of 4000 m was chosen as the threshold for analysis (Figure 5).

**Figure 6:**
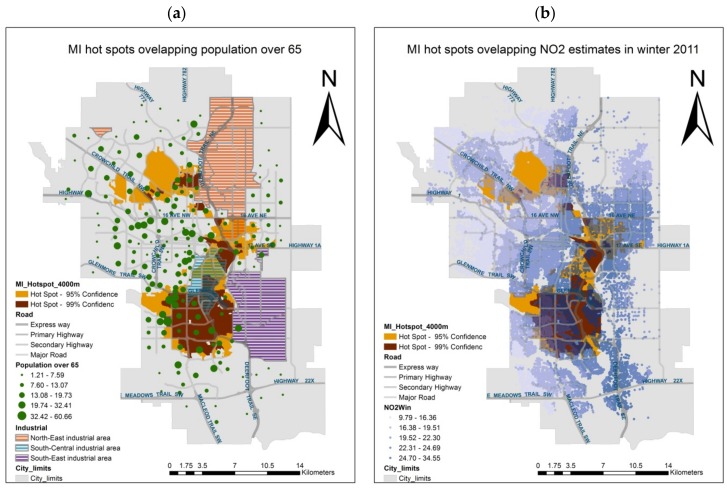
MI hot spots overlaying (**a**) population over 65, and (**b**) air pollution.

Hot spot analysis (Figure 6) over a distance threshold of 4000 m suggests that, for the overall MI population, the most significant MI hot spot (Figure 6a) is found in the south, crossing over Southwest Calgary and Southeast Calgary. It is located west of the South-East industrial area, and the north of the hot spot overlaps the south of the South-Central industrial area. The second hot spot is identified north of the south-central hot spot, between the North-East and South-Central industrial area. The third north hot spot is on the west side of north-east industrial area. Communities in these hot spots have moderately large total populations, with a high proportion of people over 65. In addition, these hot spots are located in communities with large numbers of commuters using motor vehicles as daily modes of transportation, as well as in the east of Calgary and along some of the major roads, that is, overlapping the most highly polluted areas of the city (Figure 6b).

#### Spatial Analysis for Age and Gender Specific Groups

**Figure 7:**
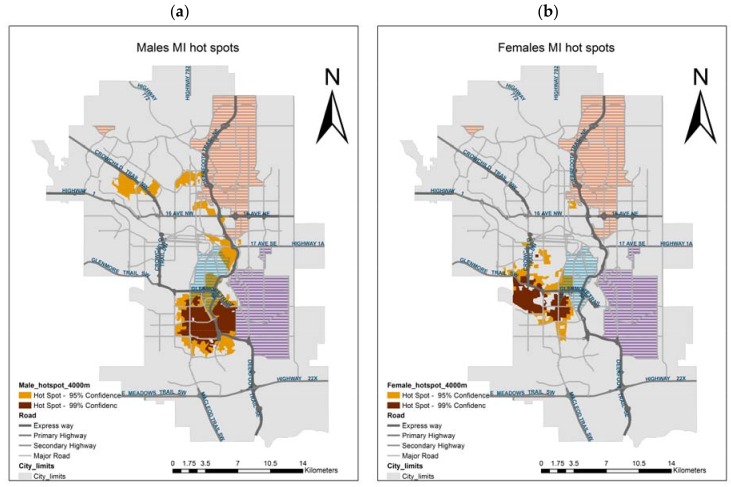
MI hot spots of (**a**) males, and (**b**) females.

The male hot spots largely coincide with those identified for overall MI population; indeed, the MI male population contributes 73% to the MI total population. The single significant hot spots for females lie west of the overall/male hot spot (Figure 7).

**Figure 8:**
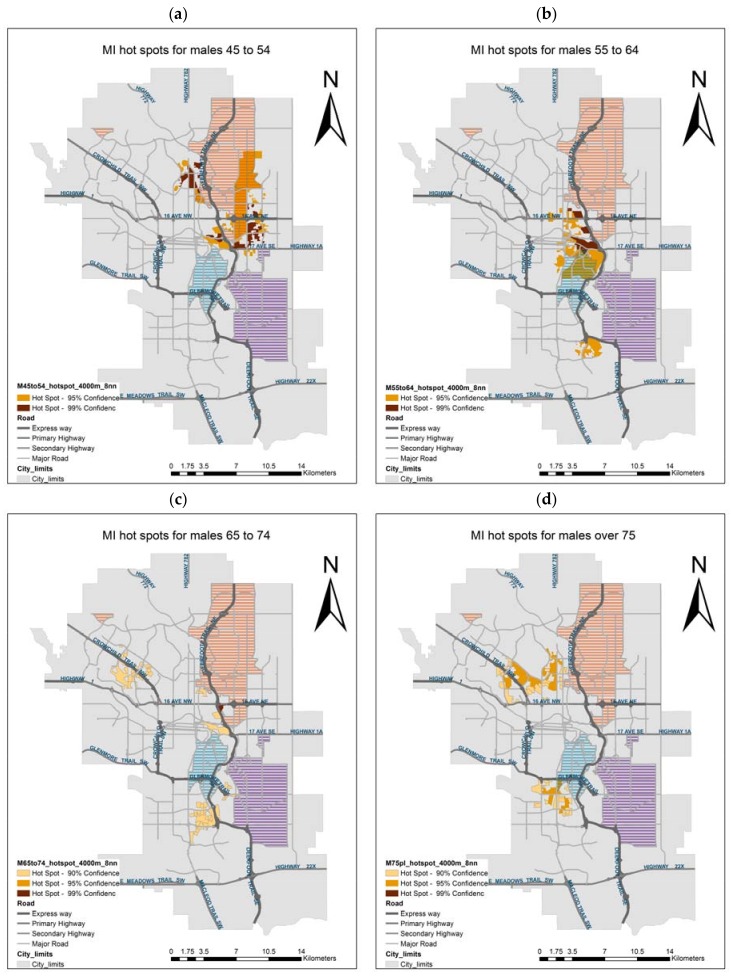
MI hot spots of males (**a**) 45 to 54, (**b**) 55 to 64, (**c**) 65 to 74, and (**d**) over 75.

In addition to the gender difference in spatial patterns, different age groups show a different location of MI hot spots. In general, the pattern of incidence for most male age groups corresponds to the three hot spots observed in Figure 7a. More specifically, hot spots for males 45 to 54 are detected in an eastern area, partially overlapping the north industrial area and next to the Calgary international airport (Figure 8a). For males 55 to 64, the hot spot lies east of the downtown core, partially overlapping the north-east and south-central industrial areas (Figure 8b). The MI incidence for males 65 to 75 displays hot spots that are less significant and smaller in size (Figure 8c); while for males over 75, hot spots are also less significant, and located in the north and south center areas (Figure 8d). No significant hot spot is detected for males aged 20 to 44, likely due to the low incidence in this group. In summary, younger males tend to develop higher MI incidence in the east area, while older males have higher MI risk in the south center.

As noted above, females account for only one third of MI cases. Likely because of these low numbers, no significant spatial hot spots are detected for any female age group based on the 4000 m neighborhood size.

## 4. Discussion

This paper presents an exploratory analysis of temporal and spatial trends in MI hospitalizations in Calgary. Even though the database consists of more than 6000 records over 10 years and about 1400 dissemination areas, the data are sparse in time and space, with a large proportion of 0s. These characteristics of the dataset limited our analysis; that is, temporal data had to be spatially aggregated, and spatial data had to be temporally aggregated. Further analysis may consider ways of conducting a simultaneous space-time analysis. Moreover, because of the above aggregation need, confounding factors were not considered explicitly in the models.

The MI hospitalization rate in males is almost three times higher than the female rate in Calgary from 2004 to 2013, consistent with other studies [11] which report that females account for about one-third of all MI hospitalizations in Canada from 1994 to 2004. The MI rate in women is positively related to age, while for men, the highest MI risk is observed in males aged 55 to 64, followed by 65 to 74 and over 75. This is consistent with the historical finding that cardiovascular disease is common among middle aged men [11]. The difference of MI risk across genders decreases with advancing age [16].

### 4.1. Seasonality

The results show that, in both males and females, the MI hospitalizations follow a seasonal pattern with peaks in both winter and summer. Winter peaks of MI incidence, or the positive effect of cold weather on MI occurrence, are consistently reported in the literature [39]. However, our study points to a summer peak, albeit somewhat less pronounced than the winter peak, which is novel, in comparison with previous studies suggesting that the seasonal pattern of MI shows a trough in summer (August) [40]. The effect of hot weather on MI varies largely across studies. Studies conducted in the tropical regions reported a possible positive association between hot dry climate and MI hospitalizations [41]. A study of the Turkish city of Kutahya shows that the increase in temperature contributes to the increase of MI cases in the same day. On the other hand, a study of Taiwan, a subtropical region with a warm climate, indicates the absence of winter and summer peaks for both males and females [42]. This inconsistency of summer MI patterns across studies may be related to the data used for the analysis, which may include cardiac mortality or mortality in general rather than only MI admissions [16]. The inconsistency may also be caused by the use of a single predictor, that is, Mean Daily Temperature for the analysis, whereas other studies suggest that the effect of hot weather conditions on MI cannot be fully appreciated with a single temperature predictor [43]. A study conducted in Italy indicates that using the traditional single predictor, Mean Daily Temperature, the results show a negative association between temperature and MI incidence. However, the same study presents an alternative analysis that uses a biometeorological approach, combining air temperature, relative humidity, and wind velocity. The results of the latter analysis indicate that hot conditions significantly increase the MI hospital admissions [43].

Overall, climatologic factors such as temperature, humidity, wind velocity, and front movement play an important role in the MI seasonality. The lowest temperature was recorded in January, which was suggested to be associated with higher MI risk. It can be explained that cold temperature results in high blood pressure variability and greater oxygen demand, hence increasing cardiac work and potential ischemic reaction especially for people with vulnerable myocardium [44,45]. While Calgary’s climate is generally cool, extreme heat conditions, with temperatures above 30 degrees Celsius, were observed with increasing frequency in the most recent summers. In addition, higher relative humidity was observed in the summer months in Calgary. The combination of hot temperature and high relative humidity may contribute to the MI peak in summer. Indeed, Panagiotakos et al. (2004) found a positive association between humidity and MI hospitalizations [15].

Regarding the gender difference in seasonality, both males and females follow a similar seasonal pattern in Calgary: the MI occurrence peaks in both summer and winter. Though the winter peak appears to be slightly higher than the summer peak, no statistical test was conducted in this study to assess the significance of the difference between the two peaks. The finding of similar seasonal patterns for both males and females is consistent with a study in Denmark reporting that hospitalization for acute MI displays similar seasonal pattern for both males and females, but the pattern is different among age groups [40]. Likewise, our study shows that all female age-stratified groups follow a consistent seasonal pattern with peaks in both winter and summer, whereas the seasonal pattern for males differs substantially across age groups. Males over 75 peaks in the winter and drops in the summer, which is consistent with the Danish study which points out that people over 80 feature a peak in December and a trough in August. Though this study did not separate males over 80 years from females over 80 years [40], given the large contribution of males to the MI population, it is reasonable to consider them as a consistent result.

Studies suggest that physical activity is negatively associated with cardiovascular diseases in both genders and physical activity in the summer can be substantially higher than in the winter [13], which may be a possible explanation for MI peaks in winter and troughs in the summer. The summer peaks of MI for females may be explained by lower physical activity, as studies indicate women usually tend to be more inactive than men. In the hot summer months, older age groups may have more difficulty in regulating body temperatures, which may expose them to higher risk of MI.

In addition to the cited Italian study [43], other literature suggests that air pollution may be a possible factor contributing to the MI seasonality [46]. Air pollutants in Calgary, e.g., PM_2.5_ and NO_2_ tend to higher in winter than in summer [22,23], and may contribute to the winter peak of MI incidence. However, ozone (O_3_) levels tend to be higher in the summer, particularly in hotter climates [43,44]. It is known that lower values of NO_2_ correspond to higher values of O_3_ [47]. Numerous studies show that higher air pollution concentration is positively associated with increased MI hospitalizations [48,49,50]. MI may be more highly associated with NO_2_ and PM_2.5_ in winter, as opposed to O_3_ in summer.

The effect of meteorological variables are likely to vary in different climates. The consistency between the Danish study [40] and our Calgary study suggests that such effects may bear some consistency across cooler, higher latitude climates. Such a geographical pattern deserves further investigation, especially for women and older age groups.

### 4.2. Long Term Linear Decreasing Trend

Overall the decline observed among most groups is encouraging; however, the rate of such decline varies across ages and genders. The decline in women is less pronounced than that in men of the same age. In both genders, people under 55 exhibit the lowest rate of decline, whereas the greatest decline is observed in males 55 to 64. The latter result is consistent with [12], which indicates the greatest decline rate in the 50 to 64 age group in Ontario, Canada. The decline of MI incidence may be associated with a declining trend in traditional risk factors for cardiovascular disease, including total cholesterol level and systolic blood pressure, as well as improvements in medical treatment. Studies suggest that risk factors and medical treatment may contribute equally to the decreasing trend [17]. According to our results, such improvements in medical treatment and risk factor reduction are more evident in the cohort exhibiting the highest MI incidence, i.e., males 55 to 64 (see Figure 2); however, lower rates of decline in younger ages, especially in women, suggest that targeted efforts should be addressed to these cohorts. Although there is a drop in MI hospitalizations in 2012, it may be too small to change the direction of the long term linear trend.

The result of this study is only for descriptive purposes, rather than decision making. Literatures suggest that multiple testing for exploratory studies is not necessary [51] or in appropriate [52]. In addition, it is not commonly used in studies applying regression models [52]. Multiple testing was not accounted for in this study.

### 4.3. Spatial Pattern and Spatio-Temporal Processes

All of the three major hotspots (Figure 6) lie in the proximity of industrial areas and major traffic corridors. As noted, the area of the southern hot spot is home to a large proportion of seniors (aged over 65). Conversely, the location of the second largest hot spot, east of downtown, is known for its low socioeconomic status, that is, relatively low income, high unemployment, low education, and a large number of people living alone [53]. The third large hot spot is quite small, located in the north, still close to industrial areas, and overlapping communities with relatively high social isolation and poverty indices [53]. All of those areas are exposed to relatively high levels of air pollution (Figure 6), suggesting that air pollution may play a role in MI occurrence across all of the hot spots areas. However, in some areas, particularly the south, pollution is likely to be mostly traffic-related, whereas in the eastern locations it is mostly of industrial origin [54]. Traffic and industrial emissions are the top two contributors to air pollution in Calgary [21]. The suggested association between air pollution and MI is in line with previous studies [55,56]. D’Ippoliti et al. (2003) found air pollution was positively associated with myocardial infarction hospitalizations in Rome, especially in the warm season (April to September) [57]. Lanki et al. (2006) suggested that increased MI hospitalizations were associated with exposure to traffic related air pollution [50]. Pollution levels exhibit seasonal patterns that are affected by meteorological processes, such as temperature and wind, as well as to the type of pollution; that is, the relative contribution of residential heating and elevated traffic volumes is higher in the winter. As a result, the southern area is exposed to greater pollution in the winter, in contrast with higher pollution levels over the eastern areas in the summer [58]. Seasonal air pollution patterns, in association with extreme temperature and low humidity, may be associated with elevated rates of MI occurrence as well as with seasonal MI peaks in different parts of the city.

As noted, this pattern of hot spots is consistent for the overall population and for males of all ages, whereas the single hot spot for females of all ages shows a slight west shift. With respect to age and gender groups, male hot spots present substantial variations: hot spots are larger and more significant for younger than for older males. With advancing age, the hot spots of MI for males appear to move from east to west and from north to south. Our results indicate that when the population is disaggregated by age and gender, the southern area exhibits only minor and less significant hotspots of older males, yet highly significant hot spots of MI in younger males occur in the industrial eastern communities, home to working class families, characterized by lower socioeconomic status, and higher industrial pollution. Little can be said about the spatial pattern of females, as the only significant hot spot is at the age aggregate level. Compared to overall and male hot spots, it present a slight north-west shift, suggesting a lesser association of female MI with industrial areas. For this hotspot, like those of older males, the disease occurrence appears to be more tightly associated with demographic factors, as it affects established communities of older median age, higher socio-economic status, and lower air pollution.

The spatial analysis was conducted on data temporally aggregated over ten years, whereas the temporal analysis was conducted on data spatially aggregated over the whole city. This constitutes perhaps the most serious limitation of our work, though the size of the database would not allow for meaningful analyses at more detailed levels. Consequently, spatiotemporal trends can be hardly inferred from this study. Nonetheless, the analysis identifies some points that may aid local authorities in defining targeted public health policies.

The decline in MI rates over the ten years in Calgary has been more rapid for males 55 to 64 and women over 75; that is, for the age groups that experience the highest rates of MI. It is quite natural to hope that the decline can be sped also for other age and gender groups, particularly for the younger ones. The spatial analysis could not identify clusters of concerns for women, and for older groups it pointed to spatial association with demographics. Therefore, the most disconcerting result is the detection of a significant hot spot of males 45 to 54 in a socioeconomically and environmentally disadvantaged area. The hot spot may be associated with the prevalence of risk factors that can be targeted by local public health policies.

The analysis yields some intriguing results. For example, hot spots on younger male MI are detected in the east, where pollution is higher in the summer; yet males tend to experience higher MI rates in the winter. Conversely, a significant hot spot of female MI was detected in the southwest, where pollution levels are higher in the winter, yet females experience peak MI incidence in the summer. These results suggest not only that MI rates are affected by numerous and interacting factors, but that the effect of these factors may exhibit local variations. Localized forms of spatial analyses can be applied to investigate these local mechanisms. Geographically weighted regression was successfully used to investigate the association of child obesity and the built environment in Calgary [59], leading to recommendations for localized targeted policies.

This study only considered explicitly air pollution, yet it pointed to the potential impact of meteorological factors on MI. Further research should be conducted in this direction, particularly in a changing climate, with increasing variability and frequency of extreme events. With more data available, a multivariate spatial and spatiotemporal analysis can aid identify the role of environmental and socioeconomic determinants of MI, indicating directions for more effective public health policies.

## 5. Conclusions

We investigated the temporal and spatial pattern of MI hospitalizations from 2004 to 2013 in Calgary, Canada. It was stratified by age and gender. Most groups show a statistically significant decreasing trend over 10 years, which is consistent with previous studies of MI trends in Canada. In general, males exhibit higher rates, as well as faster decline in MI rates. In both genders, the decline over 10 years is faster for the age groups with higher MI incidence. The slow rate of decline for younger males (45 to 54) is spatially associated with environmentally and socioeconomically disadvantaged communities. Both males and females exhibit a seasonal pattern, with MI peaks both in winter and summer. Males exhibit a higher peak in the winter, with a secondary peak in the summer, while a reversed pattern is exhibited by females. In both genders, the secondary peak becomes less prominent for older age groups. Spatial analysis suggests higher MI rates tend to cluster in communities with a larger proportion of seniors, lower socioeconomic status, and higher air pollution. Industrial pollution is more highly associated with younger ages, especially for males. The analysis helps identify areas where rates of decline are lower, along with disadvantaged socioeconomic and environmental conditions. Local public health policies can be targeted to the reduction of localized risk factors in these areas.

## Figures and Tables

**Table 1:** Linear trend model for age-gender specific MI hospitalization rates.

Trend Model	Intercept	Trend Coefficient	t Value	(*p*-Value)	R^2^
Overall MI	86.29	−0.50	−22.44	<2 × 10^−16^	0.82
Males	61.46	−0.35	−21.28	<2 × 10^−16^	0.80
45–54 Years	13.09	−0.08	−20.97	<2 × 10^−16^	0.79
55–64 Years	20.03	−0.11	−14.14	<2 × 10^−16^	0.64
65–74 Years	15.00	−0.10	−32.22	<2 × 10^−16^	0.90
Over 75 Years	15.53	−0.10	−18.06	<2 × 10^−16^	0.74
Females	24.11	−0.15	−23.32	<2 × 10^−16^	0.83
65–74 Years	5.43	−0.04	−26.93	<2 × 10^−16^	0.86
Over 75 Years	12.17	−0.09	−28.42	<2 × 10^−16^	0.88
